# Knowledge Gaps and Educational Opportunities in Congenital Toxoplasmosis: A Narrative Review of Brazilian and Global Perspectives

**DOI:** 10.3390/tropicalmed9060137

**Published:** 2024-06-20

**Authors:** David Richer Araujo Coelho, Rogerio Oliveira da Luz, Catiucia Soares Melegario, Willians Fernando Vieira, Lilian Maria Garcia Bahia-Oliveira

**Affiliations:** 1 Harvard T. H. Chan School of Public Health, Boston, MA 02115, USA; 2 School of Medicine, Federal University of Rio de Janeiro, Rio de Janeiro 21941-971, RJ, Brazil; 3 Department of Medicine, Institute of Medical Science, Federal University of Rio de Janeiro, Macaé 27930-560, RJ, Brazil; 4 Department of Anatomy, Institute of Biomedical Sciences, University of São Paulo, São Paulo 05508-000, SP, Brazil

**Keywords:** toxoplasmosis, knowledge, education, pregnant women, healthcare providers

## Abstract

Congenital toxoplasmosis is a parasitic disease caused by the transmission of the protozoan *Toxoplasma gondii* during pregnancy that can potentially cause severe consequences for the fetus or neonates. The disease disproportionately impacts the global population and is generally correlated with the Human Development Index. Despite its prevalence, there are knowledge gaps among pregnant women and healthcare providers regarding the prevention, diagnosis, and treatment of this condition. This narrative review aimed to examine the current state of knowledge of toxoplasmosis among both groups, with a focus on exploring the Brazilian and global perspectives and highlighting opportunities for enhancing education and communication. A search was conducted across five databases, and 60 studies were selected (23 in Brazil and 37 worldwide). Quantitative analysis revealed that general knowledge of toxoplasmosis among pregnant women is notably poor, with 66% of Brazilian women and 72% of women worldwide lacking sufficient understanding. Among those with some knowledge, the most recognized association is with cats (46% in Brazil and 38% worldwide), followed by raw or undercooked meat (27% in Brazil and 25% worldwide), and improperly sanitized vegetables or water (15% in Brazil and 21% worldwide). Similarly, gaps in knowledge were found among healthcare providers. Difficulty with IgG avidity test interpretation is higher in Brazil (43%) compared to worldwide (18%). The most recognized association is with cats (66% in Brazil and 74% worldwide), followed by raw or undercooked meat (49% in Brazil and 70% worldwide), and improperly sanitized vegetables or water (31% in Brazil and 32% worldwide). These findings emphasize the need for tailored local and global public health educational initiatives to enhance knowledge of toxoplasmosis among pregnant women and healthcare providers.

## 1. Introduction

Congenital toxoplasmosis is a significant public health concern caused by the transplacental transmission of the protozoan *Toxoplasma gondii* (*T. gondii*) during pregnancy [1]. The disease affects one-third of the global population, albeit unequally, and is generally associated with the Human Development Index (HDI), exhibiting a higher prevalence in Low- and Middle-Income Countries (LMICs) or regions in a given country [2,3,4]. This preventable parasitic infection can have severe consequences for the fetus or neonate, including neurological damage, visual impairment, and even fetal death [5,6]. While global efforts have been made to reduce the burden of congenital toxoplasmosis, the prevalence of this infection remains a challenge worldwide [7]. Recent data indicate a decreasing trend in its global prevalence [8,9,10]. Nonetheless, in Brazil, there has been a recent and worrisome increase in reported deaths that can be associated with congenital toxoplasmosis, necessitating a more thorough examination of this trend [11].

Preventing congenital toxoplasmosis involves adopting appropriate primary measures to reduce the risk of *T. gondii* infection during pregnancy [12]. These measures include avoiding exposure to *T. gondii* bradyzoites and sporozoites, which can result from consuming raw or undercooked meat, unfiltered water, and improperly sanitized fruits and vegetables, as well as contact with contaminated water, soil, or cat feces. These sources hold significant epidemiological relevance for *T. gondii* infection [3]. Additionally, practicing good hygiene, such as proper handwashing and food preparation techniques, can contribute to reducing the transmission risk [12]. 

Health education in the field of toxoplasmosis encompasses a range of initiatives to inform pregnant women and the general public about how to protect themselves from *T. gondii* infection. These efforts are built on extensive research and practical health strategies developed over time. A notable example is the study by Felín et al. (2022) [13], which focuses on health education about toxoplasmosis aimed at preventing its congenital form. This article evaluates and develops educational strategies specifically designed for pregnant women, considering the unique risk factors for parasite transmission in countries such as Colombia, Panama, and the United States of America (USA). It also compares the effectiveness of different educational tools, including digital ones, in disseminating knowledge of the disease. 

While education is often considered a key component in promoting preventive behaviors, with some studies indicating a positive impact of health education programs and a decrease in seroconversion rates of congenital toxoplasmosis [14,15], it is worth noting that the comprehension and assessment of the efficacy of educational initiatives in this regard still face limitations [16]. Studies have been conducted to assess the level of knowledge of toxoplasmosis in different population groups in Brazil and various countries. In all of these studies, a common characteristic is the considerable heterogeneity in the approach and structuring of the assessment of knowledge of the disease, as well as in the level of knowledge of the relevant risk factors for disease prevention [17,18,19,20].

Given the strategic role of health education during prenatal care in preventing and reducing the risk of toxoplasmosis exposure among pregnant women [21,22,23] and considering the observed heterogeneity of study proposals in the field of health education on toxoplasmosis—which reflects the complex needs in this area and underscores the importance of multidisciplinary approaches to enhance well-being—we aim to present this narrative review. It specifically focuses on analyzing studies regarding the knowledge of toxoplasmosis among pregnant women and healthcare providers, considering both Brazilian and global perspectives. Our goal is to map the understanding and identify gaps in knowledge that can potentially affect the prevention and care of toxoplasmosis in maternal health settings. This analysis is fundamental to developing targeted educational strategies that can improve the health and well-being of both pregnant women and newborns. Therefore, this review aligns with global efforts to combat congenital toxoplasmosis and highlights the importance of integrating knowledge from different areas and contexts to create effective educational strategies.

## 2. Materials and Methods

To comprehensively assess the knowledge of toxoplasmosis among pregnant women and healthcare providers, a targeted search strategy was designed. Five databases—PubMed, Embase, Biblioteca Virtual em Saúde (BVS), SciELO, and Google Scholar—were thoroughly searched using a combination of keywords, as shown in Appendix A.

In our eligibility criteria, no restrictions in terms of date of publication or study design were applied to ensure the broadest possible coverage. In terms of language, a multilingual approach was employed to achieve a comprehensive inclusion of global perspectives. However, the studies selected were limited to those published in languages where at least two members of the research team had demonstrated proficiency. The languages covered were English, Portuguese, Spanish, and French.

To be included, studies had to be related to the knowledge of toxoplasmosis among pregnant women or healthcare providers. The level and/or quality of knowledge was not considered for inclusion or exclusion. Reviews, study protocols, non-peer-reviewed articles, and studies that exclusively focused on assessing seroprevalence or other risk factors without examining knowledge of toxoplasmosis were excluded. In addition, studies that surveyed the general population, puerperal women, university students, or healthcare providers not directly involved in prenatal care were also excluded.

A careful screening of the identified articles by title and abstract and then by full text was conducted to determine whether they met the inclusion criteria. Furthermore, a manual search of the reference lists of the selected articles was performed to identify additional studies. The initial search was conducted in March 2023, with a subsequent update in March 2024.

An extraction process was designed to encompass a comprehensive range of data from the selected studies. This included the first author’s name, publication year, and location details—specifically, the city and state for studies within Brazil or the country for international studies, accompanied by the HDI at the time of the study. Additionally, the sample size for both pregnant women and healthcare providers was extracted. Information regarding pregnant women’s educational levels and healthcare providers’ years of professional experience was also recorded. Finally, key findings, such as the level of knowledge of toxoplasmosis and specific details documented by each study, were also extracted. Each study was represented on a geographical map, both in Brazil by city and state, as well as worldwide by country. We calculated the percentage of pregnant women or healthcare providers with knowledge of toxoplasmosis—general knowledge, difficulty with IgG avidity test interpretation, association with cats, association with raw or undercooked meat, and association with improperly sanitized vegetables or water—considering the number of studies analyzing each variable and the sample size of each study.

## 3. Results

### 3.1. Basic Characteristics of the Included Studies

After categorizing each study and removing duplicates, 60 studies were included (23 in Brazil and 37 worldwide). The 23 studies conducted in Brazil were clustered as follows: three from Paraná; two each from Maranhão, Minas Gerais, Pará, Rio de Janeiro, and Rio Grande do Sul; and one each from Acre, Alagoas, Goiás, Mato Grosso, Pernambuco, Piauí, Santa Catarina, Sergipe, Rio Grande do Norte, and Tocantins. The 37 studies conducted worldwide were clustered as follows: six from the USA; four each from Morocco and Nigeria; two each from Mexico, Saudi Arabia, and Tanzania; and one each from Afghanistan, Egypt, France, Iran, Iraq, Ireland, Japan, Malaysia/Philippines/Thailand, Pakistan, Poland, Romania, Sri Lanka, The Netherlands, Turkey, Vietnam, Yemen, and Zambia.

Of the 60 studies, 40 assessed the knowledge of toxoplasmosis among pregnant women, 16 among healthcare providers, and 4 among both populations. Of note, significant heterogeneity was observed in the types of questions utilized to acquire demographic data and assess the knowledge of toxoplasmosis and its preventive measures among the four groups that met the inclusion criteria proposed in this review. These varied widely, encompassing broad assessments of general awareness (such as whether participants had heard of toxoplasmosis) to more detailed inquiries into specific aspects of prevention, diagnosis, and treatment. Responses were solicited mainly through close-ended questions. Furthermore, the majority of these studies adopted a cross-sectional design and featured medium sample sizes.

**Figure 1:**
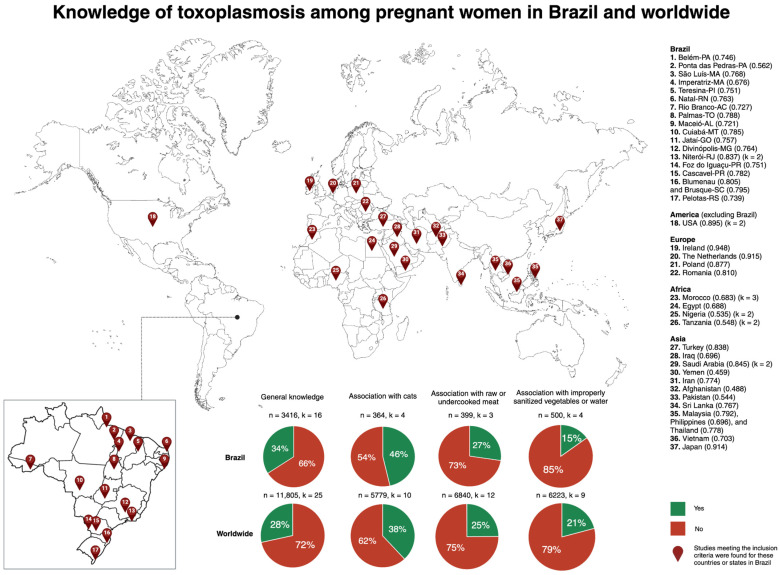
**Knowledge of toxoplasmosis among pregnant women in Brazil and worldwide.** The map illustrates the geographical distribution of studies. Locations with multiple studies are indicated by k. For these locations, the most recent Human Development Index (HDI) is provided in parenthesis. The sample size for each calculation is indicated by n. PA: Pará; MA: Maranhão; PI: Piauí; RN: Rio Grande do Norte; AC: Acre; TO: Tocantins; AL: Alagoas; MT: Mato Grosso; GO: Goiás; MG: Minas Gerais; RJ: Rio de Janeiro; PR: Paraná; SC: Santa Catarina; RS: Rio Grande do Sul; USA: The United States of America.

In the quantitative analysis, general knowledge of toxoplasmosis among pregnant women is notably poor, with 66% of Brazilian women and 72% of women worldwide lacking sufficient understanding. Among those with some knowledge, the most recognized association is with cats (46% in Brazil and 38% worldwide), followed by raw or undercooked meat (27% in Brazil and 25% worldwide), and improperly sanitized vegetables or water (15% in Brazil and 21% worldwide). Further details of these key findings and the geographical distribution of the studies are shown in Figure 1.

**Figure 2:**
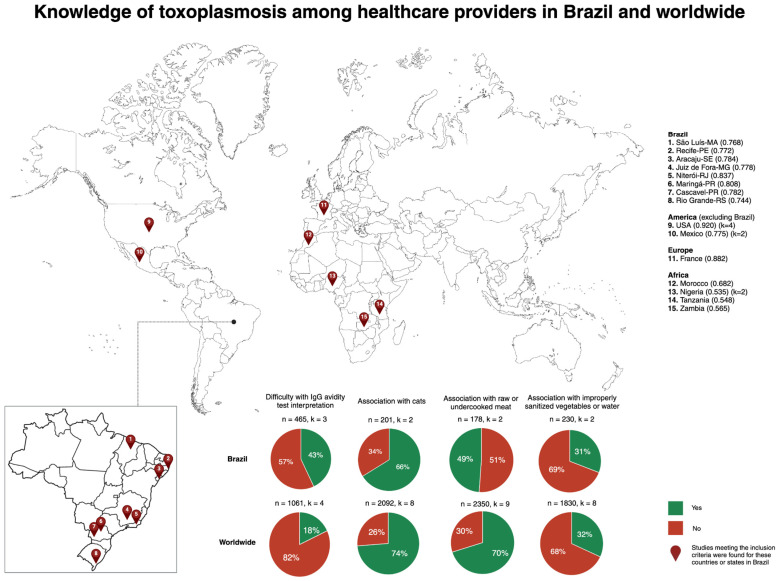
**Knowledge of toxoplasmosis among healthcare providers in Brazil and worldwide.** The map illustrates the geographical distribution of studies. Locations with multiple studies are indicated by k. For these locations, the most recent Human Development Index (HDI) is provided in parenthesis. The sample size for each calculation is indicated by n. MA: Maranhão; PE: Pernambuco; SE: Sergipe; MG: Minas Gerais; RJ: Rio de Janeiro; PR: Paraná; RS: Rio Grande do Sul; USA: The United States of America; IgG: immunoglobulin G.

Similarly, there are gaps in knowledge of toxoplasmosis among healthcare providers. Difficulty with IgG avidity test interpretation is higher in Brazil (43%) compared to worldwide (18%). The most recognized association is with cats (66% in Brazil and 74% worldwide), followed by raw or undercooked meat (49% in Brazil and 70% worldwide), and improperly sanitized vegetables or water (31% in Brazil and 32% worldwide). Further details of these key findings and the geographical distribution of the studies are shown in Figure 2.

The results are subdivided into four topics: (1) knowledge of toxoplasmosis among pregnant women in Brazil; (2) knowledge of toxoplasmosis among healthcare providers in Brazil; (3) knowledge of toxoplasmosis among pregnant women worldwide; and (4) knowledge of toxoplasmosis among healthcare providers worldwide.

### 3.2. Knowledge of Toxoplasmosis among Pregnant Women in Brazil

Multiple studies in Brazil have revealed significantly poor knowledge of toxoplasmosis and its prevention among pregnant women, with 45.2% to 93% having little to no knowledge of the disease across different studies (Supplementary Table S1) [24,25,26,27,28,29,30,31,32,33,34,35,36,37,38,39]. The most predominant piece of knowledge among those who were aware of toxoplasmosis was its association with cat contact or handling cat litter, with 17.9% to 69.8% recognizing this potential route of transmission [24,31,34,39].

Regarding other important transmission routes of toxoplasmosis, knowledge was notably lower. Only 2.2% to 28.8% of pregnant women recognized the risk of consuming raw or undercooked meat or the importance of washing hands when handling raw meat as preventive measures [24,31,33,37,39]. Knowledge of the risk posed by consuming improperly washed vegetables and fruits or drinking untreated water was also limited, with only 2.7% to 27.3% [24,31,37,39] and 7.3% to 12.2% [33,34,37] acknowledging these risks, respectively.

Finally, a few studies highlighted correlations between the level of knowledge of toxoplasmosis and demographic factors. Better knowledge was associated with increased age, higher levels of education, and the number of gestations [28,30], and a lack of knowledge was linked to lower family income and lower levels of education [29].

### 3.3. Knowledge of Toxoplasmosis among Healthcare Providers in Brazil

Studies examining the knowledge of toxoplasmosis among healthcare providers in Brazil have also highlighted varying levels of understanding (Supplementary Table S2). Notably, while 97.4% of healthcare providers correctly recognized cats as a source of *T. gondii* transmission [40] or 90.1% had some knowledge of the infection [30], there were prevalent knowledge gaps. Common misconceptions included the belief held by 51.7% that dogs could also shed the parasite [40] or 23.6% providing misguided advice about preventing transmission, such as 51.7% inaccurately cautioning against walking barefoot and 26.9% erroneously advising to avoid contact with cat urine [41].

The gaps become more pronounced when delving into specifics such as the transmission mode and diagnostic interpretations. Recognition of the risk posed by raw or undercooked meat was inconsistent, reported by only 46.5% to 67.6% of professionals [30,42], while the risks associated with waterborne sources were acknowledged by only 24.4% to 41.6% [30,41]. Misunderstandings around the IgG avidity test were common, with 18.3% to 70% of healthcare providers not recognizing its relevance in determining the timing of infection [30,40,43,44,45]. In addition, there was uncertainty surrounding the guidelines for treating pregnant women with reactive IgM [45,46].

Comparatively, physicians often outperformed other healthcare providers in their knowledge of toxoplasmosis [30,40,41,43,45]. In addition, recent graduates who had graduated in less than ten years demonstrated better knowledge of the disease [40].

### 3.4. Knowledge of Toxoplasmosis among Pregnant Women Worldwide

Multiple studies have shown that knowledge of toxoplasmosis among pregnant women worldwide varies considerably across different regions (Supplementary Table S3). Some countries exhibited notably high levels of knowledge, such as 94.7% in Yemen [47], 94.4% in Poland [48], and 75.3% in the Netherlands [49], having heard of toxoplasmosis or demonstrating basic knowledge of the disease. In contrast, others like Egypt [50], Malaysia, the Philippines, Thailand [51], Saudi Arabia [52], Sri Lanka [53], Nigeria [54,55], Tanzania [56,57], Afghanistan [58], Morocco [59,60], Vietnam [61], and Iran [62] showed alarmingly low awareness, with up to none of the pregnant women surveyed having any knowledge of the disease or its modes of transmission [54].

Despite this variance in overall knowledge, the association between toxoplasmosis and cat contact, including handling cat litter, was identified as a widely recognized risk factor. This recognition ranged from 5.2% to 77.9% [49,51,55,63,64,65,66,67], indicating a global acknowledgment of the connection between toxoplasmosis and cats. However, knowledge of other significant risk factors, such as the consumption of raw or undercooked meat and unwashed fruits and vegetables, was markedly low. The awareness of the risk posed by raw or undercooked meat varied between 2.3% and 51% [49,51,62,63,64,65,66,67,68,69,70], and for unwashed fruits and vegetables, between 5.1% and 56% [49,50,51,52,55,62,63,65,68,69], even in countries where a higher general knowledge of toxoplasmosis exists.

Lastly, the studies also revealed notable correlations between knowledge levels and demographic factors. A higher level of education was consistently associated with better knowledge of toxoplasmosis [47,48,64,68], with none of the women who had never attended school being aware of the disease [57]. Similarly, better knowledge of toxoplasmosis was correlated with multiple pregnancies [48,67,69].

### 3.5. Knowledge of Toxoplasmosis among Healthcare Providers Worldwide

Studies examining the knowledge of toxoplasmosis among healthcare providers worldwide have also exhibited varying levels of understanding (Supplementary Table S4). The majority of healthcare providers acknowledged contact with cats and the handling of cat litter as significant risk factors for toxoplasmosis transmission, with recognition rates spanning from 50% to 99.6% [71,72,73,74,75,76]. However, advice on managing this risk varied, with 52.5% of providers in the USA mistakenly recommending that keeping cats outdoors could prevent toxoplasmosis [77]. Similarly, in the USA, 29% of healthcare providers suggested avoiding all contact with cats [78]. In Nigeria, misconceptions were even more pronounced, with 36% of healthcare providers erroneously believing that humans could also shed *T. gondii* in their feces [75].

The gaps also become more pronounced when delving into specifics such as the transmission mode and diagnostic interpretations. While in certain regions, such as France, Mexico, Morocco, Nigeria, and the USA, knowledge regarding the risk associated with consuming raw or undercooked meat ranged from 60% to 100% [71,72,73,75,76,77], in other studies from Mexico and Nigeria, recognition of the same risk factor significantly dropped to 14.6% to 35% [74,79]. For waterborne sources of toxoplasmosis, France showed a high level of knowledge, with correct responses from 76.5% to 100% [80]. In contrast, awareness regarding avoiding unwashed fruits and vegetables or the importance of drinking filtered water was between 1% and 52% in Mexico, Morocco, Nigeria, and the USA [71,72,73,74,75,76,78,79]. Diagnostic interpretation concerning the IgG avidity test presented further disparities. Familiarity with the test was low at 8.8% in the USA [71], and 61.5% were unsure of what the test determines [73]. The same was found in Mexico, where 75% to 90.1% did not comprehend the significance of the IgG avidity test [72,74].

Additionally, the level of knowledge of toxoplasmosis exhibited variations across different healthcare professions. Physicians consistently demonstrated superior understanding compared to other healthcare workers, such as nurses [81], and obstetricians were noted for providing more precise preventive counseling on toxoplasmosis than their counterparts in internal medicine and family practice [78]. Notably, none of the included studies reported correlations regarding years of experience.

## 4. Discussion

Although there is limited evidence demonstrating a significant correlation between health education measures and a reduction in the seroprevalence of congenital toxoplasmosis [16], the strategic importance of education in the primary prevention of infectious diseases cannot be overlooked. Given the multifactorial nature of toxoplasmosis and its transmission dynamics within diverse cultural and socioeconomic contexts, it is essential to examine pregnant women’s and healthcare providers’ knowledge profiles to develop appropriate strategies for preventing this infection [2,3,4]. This narrative review focused on assessing knowledge of toxoplasmosis among pregnant women and healthcare providers, both within the context of Brazil and on a global scale. The quality of data across the different studies was not assessed in this review in detail. However, only peer-reviewed papers were included to ensure the credibility and reliability of the information presented. Our aim was to describe the landscape of such studies and to impartially reproduce the heterogeneity of the studies that met the established inclusion criteria.

While high-income countries have observed a decrease in the prevalence of toxoplasmosis in recent decades [8,9,10], Brazil has experienced a heterogeneous increase in the prevalence of the disease with varying impacts across different regions of the country [11]. The discussion will be subdivided into three topics for a better understanding and exploration of the inherently different aspects between the investigated groups: (1) knowledge of toxoplasmosis among pregnant women in Brazil and worldwide; (2) knowledge of toxoplasmosis among healthcare providers in Brazil and worldwide; and (3) additional considerations in toxoplasmosis education and study limitations.

### 4.1. Knowledge of Toxoplasmosis among Pregnant Women in Brazil and Worldwide

Our review highlights a marked disparity in the knowledge of toxoplasmosis among pregnant women in Brazil in comparison to those in higher HDI countries, such as the Netherlands and Poland [48,49]. In Brazil, the percentages of pregnant women reporting some knowledge of toxoplasmosis are notably low [24,25,26,27,28,29,30,31,32,33,34,35,36,37,38,39]. This gap is not merely an issue of health literacy but is deeply entwined with socioeconomic factors, where lower education and income levels correlate with a lack of disease awareness [28,29,30]. This situation is alarming given the high prevalence of toxoplasmosis in Brazil, reaching up to 80% in some areas [4], and the presence of aggressive parasite *T. gondii* strains [82,83]. The consequences of contracting toxoplasmosis during pregnancy can be severe, including fetal infection and subsequent complications such as intrauterine growth restriction, prematurity, cerebrospinal fluid abnormalities, and retinochoroiditis [5,84]. These complications can have long-lasting effects on the child’s health, making it essential for pregnant women to have the proper knowledge of prevention.

In a global context, the variability in toxoplasmosis knowledge among pregnant women is equally concerning [47,48,49,50,51,52,53,54,55,56,57,58,59,60,61,62,63,64,65,66,67,68,69,70,85,86]. While some nations have made commendable strides in educating their populations about toxoplasmosis—evidenced by higher awareness levels in countries with robust public health infrastructures, such as Austria, France, Poland, Slovenia, and other European countries [14,87,88,89,90]—many regions still face substantial challenges in bridging the knowledge gap. A global variability in awareness highlights the fundamental role of public health policies and education systems. It is imperative to recognize that enhancing the knowledge of toxoplasmosis transcends the dissemination of information. It requires the implementation of universal hygiene and food safety measures while also considering those that are culturally and socioeconomically sensitive, addressing the unique challenges faced by different communities. The need for targeted educational programs is particularly acute in regions with low HDI scores, where the combination of limited access to health information and the lack of comprehensive public health initiatives exacerbates the risk of toxoplasmosis.

**Figure 3:**
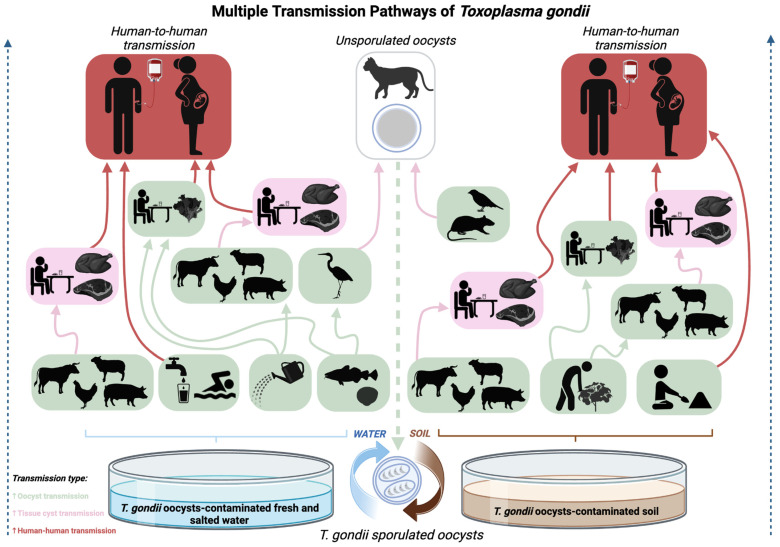
**Multiple transmission pathways of *Toxoplasma gondii*.** This illustration provides a detailed overview of the multiple transmission pathways of *Toxoplasma gondii* (*T. gondii*), encompassing environmental, foodborne, and direct human-to-human transmission within the context of the parasite’s life cycle. Initiation of the transmission cycle occurs with the release of unsporulated (non-infective) oocysts in the feces of cats (felines) after they become infected by ingesting bradyzoites from their prey. Subsequently, the oocysts sporulate (becoming infective with eight sporozoites within each oocyst) in the environment, and the contamination of soil and water sources occurs, persisting infective for a long time. The diagram specifies various transmission vehicles and infection mechanisms. Blue arrow delineates the pathway from *T. gondii*-contaminated fresh and salted water to potential hosts, while brown arrow shows the pathway from *T. gondii*-contaminated soil to potential hosts. Green compartments represent diverse infection routes and scenarios resulting from ingesting sporulated oocysts. Pink compartments detail transmission via ingestion of bradyzoites within tissue cysts. Red compartments illustrate direct human-to-human transmission routes, including vertical (congenital) transmission, blood transfusions, and organ transplantation processes. Each color-coded pathway provides an insight into the complex nature of *T. gondii*’s transmission cycle, emphasizing the distinct routes through which the parasite can infect its hosts. This figure was adapted from Bahia-Oliveira et al. (2017) [3].

It is worth noting that, while some countries with low HDI reported that a high percentage of pregnant women have heard of toxoplasmosis, such as Yemen [47], the understanding of specific preventive behaviors remains superficial, often limited to associations with cats and cat litter handling. Therefore, educational initiatives should not only aim to inform but also empower pregnant women with the knowledge and resources needed to mitigate their risk of infection effectively. By focusing on the multifaceted nature of toxoplasmosis transmission—including less commonly known routes such as the consumption of undercooked meat and particularly untreated water, as well as poorly sanitized fruits and vegetables, as detailed in Figure 3—educational initiatives could significantly enhance preventive practices.

In summary, studies conducted in Brazil and worldwide revealed significant variations in knowledge of toxoplasmosis among pregnant women. This scenario underscores the need for universal and tailored public health educational programs about toxoplasmosis for pregnant women, particularly in Brazil and other countries with low HDI scores. Such programs should be adapted to the socioeconomic conditions of the target population. Their primary objective must be to elevate awareness levels, encourage the adoption of preventive measures, and effectively communicate the risks and potential severe outcomes of congenital toxoplasmosis. By doing so, these initiatives could play a pivotal role in safeguarding not only the health of pregnant women but also that of their future children. The emphasis should be on creating educational content that is accessible, relatable, and actionable, ensuring that pregnant women from different socioeconomic backgrounds have the knowledge and resources needed to protect themselves and their unborn children from this preventable disease.

### 4.2. Knowledge of Toxoplasmosis among Healthcare Providers in Brazil and Worldwide

Our review highlights gaps in the knowledge of toxoplasmosis among healthcare providers in Brazil [30,40,41,42,43,44,45,46], echoing the patterns observed worldwide [57,71,72,73,74,75,76,77,78,79,80,81]. While there is an established general knowledge of the disease, with a substantial majority correctly identifying cats as a transmission source for *T. gondii*, there are important misconceptions, such as the misattributed role of dogs or humans in shedding oocysts or the overemphasis on avoiding all contact with cats or keeping them outdoors [40,75,77].

**Figure 4:**
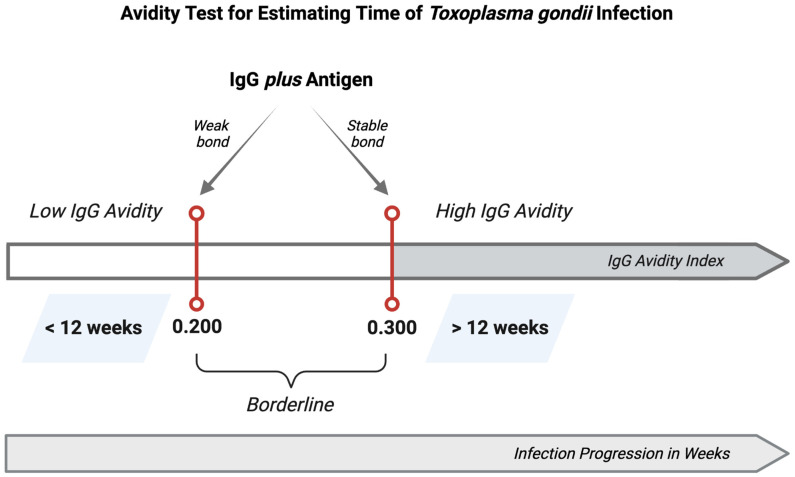
**Avidity test for estimating time of *Toxoplasma gondii* infection.** The avidity test is a commercially available enzyme-linked immunosorbent assay (ELISA) test based on the natural biological process of antibody (IgG) affinity maturation—a trait of B lymphocyte evolution into plasma cells. This maturation involves random genetic mutations in antibody genes, selecting B cells with a higher affinity for a specific antigen and producing antibodies with enhanced binding efficiency. Over time, these processes collectively refine the immune response, yielding antibodies with progressively stronger antigen-binding capabilities. In the context of *Toxoplasma gondii* infection, this test distinguishes between antibodies with low avidity—typically present in patient sera within the initial 12 weeks of infection and antibodies with higher avidity, which start to appear after 12 weeks of infection, indicating the immune response time progression and the establishment of the chronic infection. The avidity index quantifies this binding capacity, contrasting low avidity antibodies found during the acute phase of infection with high avidity antibodies of chronic infections. This figure and its underlying concepts are adapted from the VIDAS^®^ TOXO IgG Avidity test specifications [91].

In addition, disparities in knowledge of the modes of transmission further complicate the landscape. While healthcare providers in several regions are adept at identifying the risks of consuming raw or undercooked meat [30,42,71,72,73,74,75,76,77,79], they display less consistency in recognizing waterborne sources and the importance of sanitization practices for vegetables and fruits, especially in Brazil and countries with lower HDI scores [30,40,41,72,74,75,76,81]. These inconsistencies underscore a fragmented approach to education that may leave significant aspects of toxoplasmosis transmission unaddressed. Another important aspect consistent in most of the studies—both within the Brazilian context and worldwide—was the difficulty in interpreting the IgG avidity test results [30,40,43,44,45,71,72,73,74], as shown in Figure 4. These gaps indicate an urgent need for targeted educational interventions.

Moreover, healthcare providers’ knowledge of toxoplasmosis significantly varies across types of professionals, specialties, and years of experience. Physicians generally outperformed nurses and other healthcare practitioners [30,40,41,43,45,81]. In the realm of prenatal care, obstetricians showed a commendable aptitude for providing nuanced preventive counseling compared to internists and family physicians, and professionals with less than ten years of graduation tended to have a more comprehensive understanding of the disease [40,78].

These findings underscore the necessity for ongoing education and training for healthcare providers, not only in Brazil but also globally. Addressing these knowledge gaps is vital for effective toxoplasmosis management and prevention. Enhancing healthcare providers’ understanding of toxoplasmosis, including knowledge of the modes of transmission, especially concerning waterborne sources, and accurate diagnostic interpretations, like the IgG avidity test results, is imperative for delivering high-quality prenatal care. This effort will aid in preventing congenital toxoplasmosis and improving the health outcomes for both pregnant women and their children.

### 4.3. Additional Considerations in Toxoplasmosis Education and Study Limitations

While the epidemiological importance of *T. gondii* reinfections remains uncertain, their occurrence is acknowledged and represents a risk [3]. This risk is related to the vast genetic diversity of parasite strains, particularly in South America [92,93]. A notable instance, as highlighted by the French study conducted by Elbez-Rubinstein et al. (2009) [94], documented a case of congenital toxoplasmosis resulting from the reinfection of a previously immune woman exposed to a South American strain of *T. gondii*. This underscores the complexity of managing toxoplasmosis preventive measures education in our globalized world. Healthcare providers must be thoroughly informed about the potential for reinfection and incorporate this knowledge into their preventive advice to pregnant women, particularly those in endemic areas [95]. This includes educating all pregnant women to adopt precautionary measures during gestation, irrespective of their immune status.

In parallel with educational efforts, it is crucial to enhance sanitary measures that ensure the provision of water and food free from infective forms of *T. gondii*, namely oocysts and cysts [95]. The effectiveness of educational measures in preventing toxoplasmosis can be significantly amplified when coupled with improvements in the sanitation infrastructure. This approach is particularly relevant in regions with lower HDI scores, where providing safe water and food can be challenging. By addressing both educational and sanitary needs, we can more effectively combat the spread of toxoplasmosis and reduce its burden.

Despite fulfilling the criteria for classification as a neglected tropical disease, toxoplasmosis is notably absent from the World Health Organization’s list of such diseases [96]. This omission has potential negative impacts, particularly in countries with limited economic resources where there is a need for studies evaluating knowledge of toxoplasmosis and its seroepidemiological profile. The dearth of research contributes to a substantial gap in the scientific literature, imposing inherent limitations on our review’s capacity to comprehensively portray the global perspective on toxoplasmosis.

Educating with an emphasis on the primary prevention of toxoplasmosis poses a significant challenge for healthcare providers, given the variety of transmission routes, as illustrated in Figure 3, and the diversity of geographical, social, and cultural contexts in Brazil and worldwide. Our review has identified significant gaps in the knowledge of toxoplasmosis among both pregnant women and healthcare providers, a finding that is particularly pronounced in Brazil and extends globally. Insufficient knowledge of the modes of transmission among healthcare providers may impair the effectiveness of their guidance for preventing primary infection. Additionally, a lack of understanding regarding the epidemiological significance and parameters governing reinfection, especially the impact of the genetic diversity of *T. gondii* strains in South America on the immunity of pregnant women, can escalate risks, hindering the implementation of appropriate preventive strategies.

While no published studies have conclusively demonstrated the efficacy of educational interventions in toxoplasmosis prevention through statistical analysis, it is widely accepted that comprehensive knowledge is vital for successful prevention. Despite the formidable challenges posed by the disease’s multifaceted transmission, including cultural, social, and environmental factors, the importance of education in communicating transmission risks should not be underestimated. We advocate for continued and enhanced educational efforts as they are essential for protecting the health of pregnant women and their unborn children. These efforts constitute crucial steps in improving public health outcomes related to toxoplasmosis, both in Brazil and globally.

This narrative review has intrinsic limitations related to its nature that warrant consideration. First, the scope of our language inclusion was restricted to languages in which at least two of our team members are proficient (English, Portuguese, Spanish, and French). Moreover, the variable methodologies used to assess knowledge across studies introduced challenges for a detailed quantitative synthesis. Nevertheless, our primary aim was to identify and highlight gaps in knowledge that could inform improvements in healthcare delivery and implementation of public health policies. Additionally, the review’s focus on pregnant women and healthcare providers—excluding other groups such as puerperal women and university students—and the fact that many studies concentrated on seroprevalence without assessing knowledge of toxoplasmosis might have narrowed the representativeness of our findings within the Brazilian context and worldwide, especially in other countries from South America and Africa. Despite these limitations, this review is unique in its comparative scope, synthesizing information from 60 studies to shed light on knowledge gaps among pregnant women and healthcare providers, with a particular focus on Brazil—a country with a significant burden of toxoplasmosis.

## 5. Conclusions

This narrative review highlighted significant gaps in the knowledge of toxoplasmosis among both pregnant women and healthcare providers, spanning Brazilian and global contexts. Particular challenges are faced in areas with lower HDI, where educational and income limitations negatively impact knowledge of preventive measures for the disease. The heterogeneity observed in the methodologies of the analyzed studies points to the need for more standardized approaches in future research to allow for more precise comparative analyses. In Brazil, the lack of knowledge of toxoplasmosis among pregnant women, especially those of lower educational and socioeconomic levels, underscores the urgent need for targeted educational programs that consider local specificities. Globally, the variability in disease knowledge suggests that public health education initiatives, such as those observed in Poland, could be effective in increasing awareness and preventing toxoplasmosis among pregnant women. For healthcare providers, the difficulty in interpreting avidity tests and the lack of knowledge of the preventive aspects of the disease emphasize the need for ongoing training and education tailored to local realities but also informed by global guidelines where applicable. This is crucial for improving diagnosis, treatment, and counseling for pregnant women, positively impacting maternal and neonatal health outcomes. Therefore, this review highlights the importance of developing and implementing educational strategies adapted to cultural, social, and economic realities while also recognizing and addressing the need for universal knowledge on the prevention, diagnosis, and treatment of toxoplasmosis. Local and global initiatives should be complementary, ensuring that both pregnant women and health providers are equipped with the necessary knowledge to effectively prevent toxoplasmosis.

## Supplementary Materials

The following supporting information can be downloaded at: https://www.mdpi.com/article/10.3390/tropicalmed9060137/s1, Table S1: Knowledge of Toxoplasmosis among Pregnant Women in Brazil; Table S2: Knowledge of Toxoplasmosis among Healthcare Providers in Brazil; Table S3: Knowledge of Toxoplasmosis among Pregnant Women Worldwide; Table S4: Knowledge of Toxoplasmosis among Healthcare Providers Worldwide. References [97,98] are cited in Supplementary Materials.

## Appendix A. Full Search Strategy Used for Databases

PubMed	
#1	(toxoplasma[Mesh] OR toxoplasmosis[Mesh] OR “toxoplasmosis, congenital”[Mesh] OR “toxoplasma*”[tiab])
#2	(pregnancy[Mesh] OR “pregnant women”[tiab] OR “health personnel”[Mesh] OR “healthcare providers”[tiab])
#3	(knowledge[Mesh] OR knowledge[tiab] OR awareness[Mesh] OR awareness[tiab] OR information[tiab])
	#1 AND #2 AND #3
Embase	
#1	‘toxoplasmosis’/exp OR ‘Toxoplasma’/exp OR ‘congenital toxoplasmosis’/exp
#2	‘pregnancy’/exp OR ‘pregnant woman’/exp OR ‘health care personnel’/exp OR (‘healthcare provider*’):ab,kw,ti
#3	‘knowledge’/exp OR ‘awareness’/exp OR (information):ab,kw,ti
	#1 AND #2 AND #3 AND [embase]/lim
BVS	
#1	(toxoplasma OR toxoplasmosis OR “congenital toxoplasmosis”)
#2	(pregnancy OR “pregnant women” OR “health personnel” OR “healthcare providers”)
#3	(knowledge OR awareness OR information)
	#1 AND #2 AND #3
SciELO	
#1	(toxoplasma OR toxoplasmosis OR “congenital toxoplasmosis”)
#2	(pregnancy OR “pregnant women” OR “health personnel” OR “healthcare providers”)
#3	(knowledge OR awareness OR information)
	#1 AND #2 AND #3
Google scholar	
#1	(“pregnancy” OR “pregnant women” OR “health personnel” OR “healthcare providers”)
#2	(“knowledge about toxoplasmosis” OR “awareness of toxoplasmosis” OR “information on toxoplasmosis”)
	#1 AND #2
