# Supplementary material for: Knowledge Gaps and Educational Opportunities in Congenital Toxoplasmosis: A Narrative Review of Brazilian and Global Perspectives
Source: Trop Med Infect Dis. 2024 Jun 20;9(6):137. doi: 10.3390/tropicalmed9060137 (PMC11209368; doi:10.3390/tropicalmed9060137)
Supplement: Supplementary file 1 [file tropicalmed-09-00137-s001.zip › tropicalmed-2943770-supplementary.pdf]

Supplementary Table S1. Knowledge of Toxoplasmosis among Pregnant Women in Brazil.

Authors (Year)	City-State (HDI)	Number of Pregnant Women	Education	Key Findings
Cademartori et al. (2008) [24]	Pelotas-RS (0.739)	425	Not informed	64.9% had no knowledge of toxoplasmosis. Among those who were aware of the disease, 69.8% associated it with cat contact, 27.5% with consuming raw or undercooked meat, and 2.7% with consuming raw vegetables.
Barbosa et al. (2009) [25]	Natal-RN (0.763)	190	59.5% had between 5 and 10 years in school	69.5% had no knowledge of toxoplasmosis.
Fonseca et al. (2012) [26]	Divinópolis-MG (0.764)	200	64.5% had completed high school	93% had little or no knowledge of toxoplasmosis.
Contiero-Toninato et al. (2014) [43]*	Cascavel-PR (0.782)	330	Not informed	There was a general lack of knowledge of preventive measures, especially regarding the importance of washing vegetables and fruits before consumption (22.4% of incorrect answers) and drinking treated or boiled water (31% of incorrect answers).
Rodrigues et al. (2015) [27]	Teresina-PI (0.751)	64	70% had completed high school	56% had no knowledge of toxoplasmosis, and among them, 22% did not know about its modes of transmission.
Moura et al. (2016) [28]	Niterói-RJ (0.837)	405	28.9% had completed high school	57.3% had no knowledge of toxoplasmosis. Better knowledge was associated with increased age, higher levels of education, and number of gestations.
Costa et al. (2017) [29]	Belém-PA (0.746)	307	Not informed	76.9% had no knowledge of toxoplasmosis, and lack of knowledge was related to a lower family income and lower levels of education.
Sousa et al. (2017) [44]*	São Luís-MA (0.768)	15	Not informed	Pregnant women had little knowledge of toxoplasmosis.
Moura et al. (2017) [30]*	Niterói-RJ (0.837)	500	28.2% had completed high school	Only 45.2% had heard of toxoplasmosis. Better knowledge was associated with higher levels of education.
Moura et al. (2019) [31]	Imperatriz-MA (0.676)	239	41.8% had completed high school	55.6% had no knowledge of toxoplasmosis. Among those who were familiar with the condition, 28.3% reported it was caused by cats, 17.9% classified as a disease from cats, and only 5.6% said it could be transmitted through contaminated food.
Sousa et al. (2019) [32]	Rio Branco-AC (0.727)	70	60% had completed high school	51.4% had no knowledge of toxoplasmosis modes of transmission.
Souza et al. (2019) [33]	Foz do Iguaçu-PR (0.751)	82	Not informed	79.2% had no knowledge of toxoplasmosis. Only 22% reported it could be transmitted through contaminated food, and 7.3% through contaminated water.
Sampaio et al. (2020) [34]	Jataí-GO (0.757)	64	Not informed	53% did not know that toxoplasmosis could be vertically transmitted from mother to child, and 86% failed to identify all

				listed preventive methods. While 36% answered that the disease could be transmitted through contact with cat feces, only 14.7% recognized the risk of consuming improperly washed vegetables.
Morais et al. (2020) [35]	Ponta das Pedras-PA (0.562)	555	Not informed	85.2% had no knowledge of toxoplasmosis transmission.
Acioli et al. (2020) [36]	Maceió-AL (0.721)	20	45% had completed a secondary level of education	75% had no knowledge of toxoplasmosis.
Watanabe et al. (2020) [37]	Cuiabá-MT (0.785)	205	36.6% had completed high school	While 70% had some knowledge of toxoplasmosis, only 28.8% identified the risk of transmission through consuming raw or undercooked meat, 27.3% through consuming improperly washed vegetables, and 12.2% through drinking tap water.
Kohler et al. (2022) [38]	Blumenau and Brusque-SC (0.805 and 0.795, respectively)	109	36.7% had completed high school	69.2% (Blumenau) and 71.9% (Brusque) had no knowledge of toxoplasmosis or had only heard of it.
Lima et al. (2022) [39]	Palmas-TO (0.788)	45	71.1% had completed high school	47% had no knowledge of toxoplasmosis, while 29% had limited knowledge. Avoiding direct contact with cats was the most cited preventive method (26.6%), followed by avoiding the consumption of undercooked meat (20%) and avoiding direct contact with soil and/or cat litter boxes (17.7%). Only 2.2% reported the importance of washing hands when handling raw meat.

*Study with pregnant women and healthcare providers. HDI: Human Development Index in the year the study was published [97]; RS: Rio Grande do Sul; RN: Rio Grande do Norte; MG: Minas Gerais; PR: Paraná; Pi: Piauí; RJ: Rio de Janeiro; PA: Pará; MA: Maranhão; AC: Acre; GO: Goiás; AL: Alagoas; MT: Mato Grosso; SC: Santa Catarina; TO: Tocantins.

Supplementary Table S2. Knowledge of Toxoplasmosis among Healthcare Providers in Brazil.

Authors (Year)	City-State (HDI)	Participants	Years of experience	Key Findings
Da Silva et al. (2011) [40]	Juiz de Fora-MG (0.778)	112 healthcare providers (61 physicians and 56 nurses)	Not informed	While 97.4% correctly identified cats as the animal shedding the parasite in feces, 51.7% erroneously believed dogs could also eliminate oocysts. Most significant misconceptions were concerning the education of non-immune pregnant women about consuming raw vegetables without emphasizing the necessity of proper sanitization to eliminate oocysts. 18.3% answered that IgG avidity test does not relate to time of infection. Physicians had higher scores in diagnostic and clinical issues. Healthcare providers who scored higher had graduated in less than 10 years.
Branco et al. (2012) [45]	Maringá-PR (0.808)	212 healthcare providers (66 physicians, 35 nurses, and 111 nurse assistants)	Not informed	88.7% were unsure about infectious forms of *T. gondii*. Physicians had higher scores compared to other professionals. However, nearly 70% of physicians were unaware of the need for an avidity test when both IgM and IgG positive are reagent for toxoplasmosis. 39.4% were uncertain about the gestational age at which the disease can be contracted, and 15.1% were unaware of prevention guidelines for at-risk pregnant women.
Gonçalves et al. (2013) [46]	Rio Grande-RS (0.744)	29 healthcare providers (15	Average of 17 years after graduation	Only 6.9% responded that pregnant women with a reactive IgM test should be treated with spiramycin, while 27.5% either

		physicians and 14 nurses)		responded with “Does not know” or selected “None” for this question.
Contiero-Toninato et al. (2014) [43]*	Cascavel-PR (0.782)	80 healthcare providers (44 nurses and 36 physicians)	Not informed	Professionals had difficulty interpreting the avidity test results. Physicians had better knowledge of toxoplasmosis, including transmission risks, and nurses displayed less knowledge.
Sousa et al. (2017) [44]*	São Luís-MA (0.768)	15 nurses	5-20 years of experience after graduation	Nurses demonstrated some knowledge of toxoplasmosis but were found to have limited knowledge regarding its prevention and interpreting the avidity test results.
Moura et al. (2017) [30]*	Niterói-RJ (0.837)	141 healthcare providers (23 physicians, 22 nurses, 36 nursing technicians, and 60 community health agents)	1-10 years of experience after graduation	While 90.1% claimed to have some knowledge of toxoplasmosis, only 46.5% correctly identified its transmission through consuming raw or undercooked meat and 24.4% through consuming vegetables and fruits without proper hygiene. Additionally, 22.2% responded that the IgG avidity test does not determine infection time. Physicians scored higher than other professionals.
Inagaki et al. (2021) [41]	Aracaju-SE (0.784)	89 healthcare providers (69 nurses and 26 physicians)	Average of 18.5 years after graduation	Only 41.6% correctly advised against consuming untreated water. 51.7% incorrectly advised not walking barefoot, and 26.9% erroneously recommended avoiding contact with cat urine. Physicians had better knowledge compared to nurses.
Santos et al. (2023) [42]	Recife-PE (0.772)	37 healthcare providers (3 physicians, 20 nurses, 3 nursing technicians, and 11 community health agents)	Not informed	70.3% identified contaminated food as a potential source of toxoplasmosis transmission, while 67.6% acknowledged that consuming raw or undercooked meat could also be a transmission vector.

*Study with pregnant women and healthcare providers. HDI: Human Development Index in the year the study was published [97]; MG: Minas Gerais; PR: Paraná; RS: Rio Grande do Sul; MA: Maranhão; RJ: Rio de Janeiro; SE: Sergipe; PE: Pernambuco.

Supplementary Table S3. Knowledge of Toxoplasmosis among Pregnant Women Worldwide.

Authors (Year)	Country (HDI)	Number of Pregnant Women	Education	Key Findings
Jones et al. (2003) [63]	USA (0.893)	403	31% had completed high school	While 48% had heard of toxoplasmosis, only 7% were aware of having been tested for the disease. Although 61% recognized that infected cats could shed the parasite in their feces and 60% recognized the risk posed by handling cat litter, there was uncertainty about preventive measures: 56% were unsure about avoiding unwashed vegetables, and 50% had doubts regarding the consumption of raw or undercooked meat.
Ogunmodede et al. (2005) [64]	USA (0.895)	322	46% had completed a high school education or less	While 42% had heard of toxoplasmosis and 62% knew about the risk of contracting the infection by changing cat litter, only 26% recognized the risk of eating raw or undercooked meat. A higher level of education was associated with better knowledge about toxoplasmosis.

El Deeb et al. (2012) [50]	Egypt (0.688)	323	81.1% had literacy skills	87.9% had no knowledge of toxoplasmosis transmission modes.
Amin et al. (2013) [65]	Saudi Arabia (0.845)	234	37.2% had completed a secondary education	While 39.1% recognized cats spreading the disease, only 15.3% and 7.6% reported that consuming undercooked meat or eating unwashed fruits and vegetables could increase the risk of toxoplasmosis, respectively.
Pereboom et al. (2013) [49]	The Netherlands (0.915)	1097	56.9% had completed high school	75.3% had heard of toxoplasmosis. While 77.9% and 74.3%, respectively, identified avoiding changing the cat's litter box and using gloves when gardening as preventive methods, only 48.2% and 48.1%, respectively, acknowledged consuming unwashed vegetables and raw or undercooked meat as risk factors.
Popa et al. (2013) [70]	Romania (0.810)	267	50% had completed college	While 68.7% had heard of toxoplasmosis, only 35% recognized consuming raw or undercooked meat as a risk factor.
Andiappan et al. (2014) [51]	Malaysia (0.792)<br>Philippines (0.696)<br>Thailand (0.778)	2598	51.7% completed a secondary level of education	Only 11% had information about toxoplasmosis. Only 3.5% were conscious of undergoing screening for the infection. Only a minor proportion was informed that *T. gondii* could be present in the feces of infected cats (19.4%), raw or undercooked meat (11%), and untreated water (6.9%).
Morioka et al. (2014) [85]	Japan (0.914)	343	Not informed	While 58% had some knowledge of toxoplasmosis, only 22% knew about the risk of consuming raw or undercooked meat.
Elsafi et al. (2015) [52]	Saudi Arabia (0.845)	400	32% had completed high school	75.5% were unfamiliar with toxoplasmosis and its associated risk factors. Only 20% knew the risk of consuming unwashed vegetables and fruits.
Chandrasena et al. (2016) [53]	Sri Lanka (0.767)	293	Over 70% had completed a basic education	Only 4.4% were aware of toxoplasmosis.
Alfadly et al. (2017) [47]	Yemen (0.459)	150	54% had completed a primary education	While 94.7% had heard of toxoplasmosis, 38.7% incorrectly responded that the disease could be acquired by the superficial touch of a cat, and 43% did not know that gardening without gloves could be a risk factor. Better knowledge was associated with higher levels of education.
Dairo et al. (2018) [54]	Nigeria (0.531)	377	67.9% had completed a secondary education	None of the pregnant women surveyed had any knowledge of toxoplasmosis or its modes of transmission.
Paul et al. (2018) [56]	Tanzania (0.538)	254	74.8% had completed a primary education	89.4% had never heard of toxoplasmosis, and 91.3% did not know about the modes of transmission.
Smereka et al. (2018) [48]	Poland (0.877)	465	53.5% had completed high school	94.4% demonstrated a basic knowledge of toxoplasmosis. Factors such as higher educational attainment and the number of children positively correlated with enhanced knowledge about *T. gondii* and the symptoms of toxoplasmosis.
Al-Hellaly et al. (2019) [66]	Iraq (0.696)	98	57.14% had completed primary education	While 60.20% were informed about toxoplasmosis and 64.29% recognized that cat feces could spread the disease, only 51% were aware of the risk of consuming raw or undercooked meat.
Basit et al. (2019) [68]	Ireland (0.948)	287	59.4% had completed a third level of education	While 49.6% had heard of toxoplasmosis, only 26.2% correctly identified not consuming raw or undercooked meat and avoiding unwashed fruits and vegetables as preventive measures. Knowledge of toxoplasmosis was significantly associated with increasing education.
Mosawi et al. (2019) [58]	Afghanistan (0.488)	431	Not informed	72.9% had no knowledge of toxoplasmosis.

Onduru et al. (2019) [57]*	Tanzania (0.548)	371	87% had completed a primary education	96% had no knowledge of toxoplasmosis. Out of the 26 women who had never attended school, all (100%) were unaware of toxoplasmosis.
Ouzennou et al. (2019) [59]	Morocco (0.683)	600	Not informed	99% had no knowledge of toxoplasmosis.
Smit et al. (2019) [61]	Vietnam (0.703)	795	Not informed	97.4% had never heard of toxoplasmosis.
Eroglu et al. (2021) [69]	Turkey (0.838)	214	31.8% had completed a secondary education	22.4% had heard of toxoplasmosis. Only 5.1% and 2.3%, respectively, identified consuming unwashed fruits and vegetables and consuming raw or undercooked meat as risk factors. Higher levels of knowledge were observed in women with multiple pregnancies compared to those with their first pregnancy.
Hamou et al. (2021) [86]	Morocco (0.683)	390	24.9% had no literacy skills	41.2% reported being exposed to information about toxoplasmosis, and 13.7% associated the disease with cats.
Boussaa et al. (2022) [60]	Morocco (0.683)	100	20% have completed a secondary level of education	62% had never heard of toxoplasmosis, and only 29% had knowledge of the preventive methods.
Gheshlaghi et al. (2022) [62]	Iran (0.774)	740	42.2% had completed high school	98.2% had never heard of toxoplasmosis, and only 29.7% and 28.9%, respectively, identified eating raw vegetables and consuming raw or undercooked meat as risk factors.
Onyinye et al. (2023) [55]	Nigeria (0.535)	250	57% had completed a tertiary level of education	Only 10.8% had heard of toxoplasmosis. Among those, only 5.2% recognized the risk of contact with infected cats and contaminated water.
Khan et al. (2023) [67]	Pakistan (0.544)	237	71.7% had no literacy skills	While 54.1% had heard of toxoplasmosis and 49.3% recognized that cat feces could spread the disease, only 23% knew that the disease could be prevented by not consuming undercooked meat. The number of children a multiparous woman had was a key factor correlated with her awareness of toxoplasmosis.

*Study with pregnant women and healthcare providers. HDI: Human Development Index in the year the study was published [98]; USA: The United States of America; *T. gondii*: *Toxoplasma gondii*.

Supplementary Table S4. Knowledge of Toxoplasmosis among Healthcare Providers Worldwide.

Authors (Year)	Country (HDI)	Participants	Years of experience	Key Findings
Jones et al. (2001) [77]	USA (0.892)	364 members of ACOG	The average length of clinical experience after completing residency was 13 years	89.8% identified not eating raw or undercooked meat as a preventive method, 86.2% advised washing hands after handling meat, and 78.7% recommended wearing gloves when changing litter. 52.5% responded that it would be better to keep a cat completely outdoors in order to prevent the disease.
Kravetz et al. (2005) [78]	USA (0.894)	49 obstetricians, 40 internists, and 13 family practitioners	Not informed	29% incorrectly recommended avoiding all contact with cats, and only 1% identified eating unwashed vegetables as a risk factor. Obstetricians were found to give more accurate preventive counseling for toxoplasmosis than internists and family doctors.

Jones et al. (2010) [71]	USA (0.913)	502 members of ACOG	Not informed	While 99.6% counseled pregnant women about cat litter to prevent toxoplasmosis, and 77.6% recommended not eating undercooked meat, only 34.2% advised washing fruits and vegetables. Only 8.8% were familiar with the IgG avidity test.
Alvarado-Esquivel et al. (2011) [72]	Mexico (0.760)	100 physicians	55% had less than 10 years of clinical experience	While 86% and 60%, respectively, identified contact with cats and consuming raw or undercooked meat as risk factors for toxoplasmosis, only 19% acknowledged ingestion of unfiltered water as a potential source of infection. In addition, 75% did not know about the IgG avidity test.
Sellier et al. (2012) [80]	France (0.882)	102 midwives	The average length of clinical experience after graduation was 18 years	Correct responses for preventive methods ranged from 76.5% to 100%, with 100% of correct responses specifically acknowledging the importance of not eating raw meat or unwashed vegetables.
Davis et al. (2015) [73]	USA (0.920)	267 members of ACOG	The average length of clinical experience after graduation was 21 years	While 95.1% and 83.3%, respectively, identified changing cat litter without gloves and eating raw or undercooked meat as risk factors for toxoplasmosis, only 33.6% recognized eating unwashed fruits or vegetables. In addition, 61.5% did not know what the IgG avidity test helped determine.
Alvarado-Esquivel et al. (2017) [74]	Mexico (0.775)	192 clinical laboratory professionals (116 chemists or biologists, 63 laboratory technicians, 3 physicians, and 10 defined as others)	77.7% had less than 10 years of clinical experience	While 50% responded that toxoplasmosis could be transmitted through contact with cats, only 14.6% recognized the risk through ingesting raw or undercooked meat, 13.5% through consuming unwashed fruits or vegetables, and 13% through ingestion of unfiltered water. 90.1% did not know what the IgG avidity test represents.
Efunshile et al. (2017) [75]	Nigeria (0.532)	522 medical doctors	48.1% had between 5 and 10 years of clinical experience	While 62% acknowledged that cats could shed *T. gondii* in their feces, 36% incorrectly believed humans could also shed the parasite. Although 69% were aware of the meat-borne risk of *T. gondii* infection, only 28% understood it could be waterborne as well.
Onduru et al. (2019) [57]*	Tanzania (0.548)	22 health workers (12 nurses, 4 medical officers, 2 maternal and child health assistants, 3 laboratory technicians, and 1 sonographer)	Not informed	Only 36% were aware of the disease and its clinical outcomes.

Efunshille et al. (2020) [79]	Nigeria (0.535)	205 health professionals (71 resident doctors, 60 nurses, 33 medical consultants, 10 laboratory scientists or scientific officers, and 31 defined as others)	37% had between 5 to 10 years of clinical experience	While 52% responded that consuming food or water contaminated with feces of infected hosts could be a potential transmission source for toxoplasmosis, only 35% correctly identified consuming undercooked meat as a risk factor.
Laboudi et al. (2020) [76]	Morocco (0.682)	96 health professionals (54 medical doctors, 19 nurses, 14 midwives, and 9 laboratory technicians)	The average length of clinical experience was 16 years	While 90.6% identified eating undercooked meat as a risk factor for toxoplasmosis and 81.3% acknowledged direct contact with cats, only 30.2 responded correctly that untreated water could be a potential source for acquiring the disease.
Daka et al. (2023) [81]	Zambia (0.565)	175 healthcare workers (85 nurses, 38 clinicians, 34 biomedical scientists, 12 pharmacy staff, and 6 defined as others)	The average length of clinical experience was 3 years	Only 26.3% had a good level of knowledge of toxoplasmosis. Nurses, compared to clinicians, were found to have a lower likelihood of possessing good knowledge of the disease.

*Study with pregnant women and healthcare providers. HDI: Human Development Index in the year the study was published [98]; USA: The United States of America; ACOG: American College of Obstetricians and Gynecologists; IgG: immunoglobulin G; *T. gondii*: *Toxoplasma gondii.*
